# Burning pain secondary to clozapine use: a case report

**DOI:** 10.1186/s12888-014-0299-3

**Published:** 2014-10-23

**Authors:** Bradley Linton, Rachel Fu, Penny A MacDonald, Hooman Ganjavi

**Affiliations:** Department of Pharmacy, London Health Sciences Centre, 800 Commissioners Road East, North Tower, Room B7-002, N6A 5W9 London, ON Canada; Brain and Mind Institute, Natural Sciences Centre, University of Western Ontario, N6A 5B7 London, ON Canada; Department of Clinical Neurological Sciences, London Health Sciences Centre, University Hospital, Rm C7-002, 339 Windermere Road, N6A 5A5 London, ON Canada; Department of Psychiatry, University of Western Ontario, 800 Commissioners Road East, Zone B, Room B8-114, N6A 5W9 London, ON Canada

**Keywords:** Clozapine, Psychosis, Adverse effects

## Abstract

**Background:**

The first of the atypical antipsychotics introduced in the 1970s, clozapine remains the most efficacious neuroleptic to this day. However, serious and potentially fatal side effects have necessitated careful regular monitoring among prescribing clinicians. Some adverse effects (e.g. ischaemic bowel) remain under recognized, while newly identified adverse effects continue to be described in the literature.

**Case presentation:**

In this report, we describe a healthy 43-year old Caucasian male who experienced onset of a full body deep burning pain several months after the onset of treatment with clozapine. The pain worsened over time, ceased with cessation of treatment, and returned soon after the patient was rechallenged.

**Conclusion:**

We describe an unusual adverse effect from clozapine treatment that has not been described elsewhere to our knowledge. We present the time course of the pain symptom, relationship to dose, associated laboratory results, and ultimately how it was dealt with and how it improved for the benefit of clinicians who may encounter it in the future.

## Background

Despite its superior efficacy [1-3], clozapine remains an underutilized medication due to serious and potentially life-threatening adverse effects [4]. Although pain has been described as an adverse effect of clozapine, it is usually in the context of cardiac effects [5], colitis [6], serositis [7], or other well-described complications of clozapine use. Here, we describe an unusual presentation of a deep, full-body burning pain that did not appear to be associated with any of the well-described adverse effects of clozapine. We describe the clinical presentation, potential mechanisms, and how this adverse effect was managed.

## Case presentation

MT, a 44 year old Caucasian male with a several year history of psychotic depression, was admitted to hospital for treatment on March 17, 2012. His medical history was significant for chronic headaches, degenerative disc disease in the cervical spine, remote knee arthroscopy, and spontaneous pneumothorax in 1996. None of these medical issues were felt to be contributing to his psychiatric presentation. MT was a smoker, but did not use other drugs or alcohol. Physical examination including screening neurological examination did not reveal any abnormalities. Up until recently, MT was living a fairly functional life. He was married with two children, previously worked in the military, and was working as a security guard until his hospitalisation. He did not appear to have any significant psychiatric difficulties earlier in his life.

MT’s mood was depressed and he met the Diagnostic and Statistical Manual of Mental Disorders, Fourth Edition (DSM-IV) for a Major Depressive Episode. He had been depressed since early 2011 and felt it was largely related to the end of his marriage. MT reported that the auditory hallucinations began several months after the onset of depression, and at a time when he was particularly isolated and the depression quite severe. MT reported that he hadn’t had hallucinations prior to the onset of his depression.

MT’s psychotic symptoms manifested as a single male voice making derogatory comments about him, and telling him to hurt other people. He was also troubled by violent imagery in his mind. He never experienced any somatic delusions or coenesthetic hallucinations. He had previously been tried on risperidone, quetiapine, olanzapine, and haloperidol. During this stay, the patient was started on clozapine (March 20, 2012) and titrated up to 500 mg daily dose prior to discharge. This was the first time the patient had a trial of clozapine. Clozapine is not an approved treatment for psychotic depression, so the treatment was considered an off-label use of the medication. At the time of discharge, the patient was also on quetiapine 50 mg four times daily and quetiapine 200 mg nightly as needed. Aside from excessive salivation and mild constipation, the patient appeared to tolerate the medications well. The patient found this regimen to be effective at controlling his auditory hallucinations and was discharged on April 13, 2012. Depression was managed with venlafaxine XR 225 mg daily.

MT claims that a few months after discharge he experienced a “burning pain” which awoke him in the middle of the night. He claims it happened once or twice per week and would last for approximately five to 10 minutes, the longest episode lasting approximately 30 minutes. The pain was described as a “head to toe, crippling burning pain” in his “bones” that sometimes caused him to writhe on the cool floor in attempt to achieve some relief. The pain progressively became more frequent and longer in duration, causing MT to discontinue clozapine between the time of August 27 and September 25 of 2012. The patient reports that after the medication was stopped, this pain did not return. The only other medication the patient was on at this time was venlafaxine XR 300 mg daily.

Interestingly, the patient was diagnosed with a stomach infection during the same time and was prescribed a course of antibiotics. The patient denied any constipation during this time. According to the patient, during this period, he was experiencing frequent bouts of “dry heaving” whenever he would consume any solid food. He stated that no food would come up during these heaving fits, but thick mucus would sometimes be expelled. MT’s blood work from April 25, 2012 to October 18, 2012 often showed elevated leukocytes and neutrophils. His leukocytes went as high as 18.8 × 10^9^/L and neutrophils 14.7 × 10^9^/L on May 30, 2012.

MT restarted the clozapine shortly after an outpatient appointment with his psychiatrist on September 25, 2012. He started taking clozapine again at 200 mg at bedtime and was titrated back up to his usual dose of 500 mg. During this re-challenge, MT experienced similar pain as he had previously and immediately stopped using clozapine.

MT tried other antipsychotics which were not effective in controlling his auditory hallucinations and stopped using medications altogether. Eventually, he returned to hospital on April 2, 2013 when he was admitted with worsening intolerable auditory hallucinations.

During this admission MT was rechallenged on clozapine since it was the only medication shown to effectively reduce the auditory hallucinations. Clozapine was initiated at 25 mg at bedtime on April 3, 2013 and titrated up to 400 mg at bedtime on April 22, 2013 when the pain returned. The patient described the pain as the same pain he experienced before, a burning, head to toe sensation that lasted approximately two to three minutes.

A clozapine level was drawn at on April 23, 2013, but was unremarkable (clozapine =612 nmol/L; norclozapine =544 nmol/L). Other bloodwork from April 23 was also unremarkable (leukocytes =8.6 × 10^9^/L; erythrocytes =5.12 × 10^12^/L; neutrophils =5.7 × 10^9^/L; lymphocytes =2.1 × 10^9^/L). However, the previous bloodwork on April 15, 2013 showed elevated leukocytes (12.0 × 10^9^) with elevated neutrophils (8.8 × 10^9^). This was an isolated circumstance of increased leukocytes during this admission. MT was also using quetiapine 100 mg every two hours when needed and olanzapine 20 mg daily at the time of the reaction.

MT’s dose of clozapine was decreased on April 23 to 350 mg at bedtime then increased again to 400 mg at bedtime on April 24 which he was able to tolerate. The bedtime dose was not further increased from 400 mg. The total dose of clozapine was increased to 500 mg, but split with 100 mg given in the morning and 400 mg given at bedtime. MT did not experience any pain on this regimen and was discharged on May 6, 2013 with instructions to increase the clozapine to 200 mg in the morning and 400 mg at bedtime.

On June 7, at an outpatient follow-up appointment, MT reported the full body burning pain sensation was once again occurring sporadically on certain nights of the week. He also reported that the symptoms of dry heaving and abdominal pain had returned as well, similar to the symptoms he previously described.

It seemed to the treating team that MT’s reaction could be concentration dependent as the reaction always occurs within hours after the patient administering his clozapine dose. This is consistent with the time to maximum concentration of clozapine which is cited as 2.5 hours (1–6 hour range) [8]. This theory led to his treating team dividing the dose while he was admitted in an attempt to prevent the reaction from occurring. While dividing the dose reduced his pain during his admission, the pain appeared to have worsened since his discharge.

MT is currently working during the nights and sometimes takes two doses within close proximity to each other due to his work schedule. These times appeared to coincide with the times MT experienced the worst pain. We encouraged MT to keep a log of when he experienced pain to better understand the pattern of occurrence.

There are also many documented cases of infections leading to increased serum concentrations of clozapine [9-12]. MT’s gastric infection could have increased the serum concentration of clozapine at the time, worsening the burning pain. The infection was treated; however, a high neutrophil count persisted throughout his treatment course in 2012. He also had an increased neutrophil count on April 15, 2013, which was just prior to the return of the burning pain during the re-challenge while admitted in hospital. It is possible that there is an association between increased neutrophils and this patient’s “deep, burning, bone pain.” The mechanism behind this reaction is unknown; however, a Naranjo score of 7 (Table 1) would suggest that clozapine is *probably* the cause of this patient’s bone pain. An elevated c-reactive protein (CRP) level in October 2012 (6.3 mg/L) is consistent with an inflammatory process. There are no other CRP levels to compare this to. Further investigations would be beneficial to determine if inflammation could be contributing to the pain response, and what type of inflammation is occurring. There are reports of polyserositis occurring with clozapine [7], but MT’s clinical picture was somewhat different given that pain was the only symptom he described. There was no evidence of fever, shortness of breath, or gastrointestinal symptoms, which are symptoms commonly described in serositis.

**Table 1:** **Naranjo score for clozapine-induced pain**

**Question**	**Answer**	**Score**
1. Are there previous *conclusive* reports on this reaction?	No	0
2. Did the adverse event occur after the suspected drug was administered?	Yes	2
3. Did the adverse reaction improve when the drug was discontinued or a *specific* antagonist was administered?	Yes	1
4. Did the adverse reaction reappear when the drug was readministered?	Yes	2
5. Are there alternative causes (other than the drug) that could have on their own caused the reaction?	Don’t know	0
6. Did the reaction reappear when a placebo was given?	Don’t know	0
7. Was the drug detected in the blood (or other fluids) in concentrations known to be toxic?	No	0
8. Was the reaction more severe when the dose was increased or less severe when the dose was decreased?	Yes	1
9. Did the patient have a similar reaction to the same or similar drugs in *any* previous exposure?	Yes	1
10. Was the adverse event confirmed by any objective evidence?	No	0
Total		7

The treating team also considered whether MT’s pain symptoms could be somatic delusions or coenesthetic hallucinations. We felt this was unlikely as MT was frustrated at the possibility of having to discontinue clozapine given that it was the medication that helped him the most. We also observed that no other side effects MT experienced from medication were unusual. MT reported common predictable side effects such as rigidity from risperidone, sedation from olanzapine, and excessive salivation from clozapine. We felt that MT’s reporting of side effects was reliable. The only convincing psychotic symptoms were the auditory hallucinations.

MT’s most recent out-patient appointment was on August 9, 2013. He reported to still be experiencing the pain, but able to tolerate it. He reported the pain was less intense than what he previously experienced. His log was beneficial as it confirmed that the pain was worse when two doses of 200 mg and 400 mg were taken close together. He is currently using 400 mg at bedtime and 200 mg in the morning. He also eliminated one weekly dose when he transitions from night shifts to day shifts to avoid two doses taken in close proximity, and this appears to have been beneficial.

## Conclusion

To our knowledge, this is the first known case of unexplained full body burning pain associated with clozapine use. Although it is not possible to say with absolute certainty that clozapine caused this symptom, the association is highly probable. The pain subsided upon cessation of the medication on two separate occasions. The pain appears to be dose-dependent and was most severe when serum levels would be highest. Finally, the pain improved when doses above 400 mg were split into lower doses. The mechanism is unknown, but may be related to an inflammatory process. Clinicians should be aware of this infrequent but significant side effect and could attempt using divided doses to minimize the severity. The seriousness of this side effect is not known; hence, careful monitoring of any patient with pain of unknown origin would be warranted.

## Consent

The patient has given his consent for this case report to be published.
